# Analysis of Acousto-Optic Figure of Merit in KGW and KYW Crystals

**DOI:** 10.3390/ma15228183

**Published:** 2022-11-17

**Authors:** Konstantin B. Yushkov, Natalya F. Naumenko, Vladimir Ya. Molchanov

**Affiliations:** Acousto-Optical Research Center, University of Science and Technology MISIS, 119049 Moscow, Russia

**Keywords:** acousto-optics, diffraction, figure of merit, monoclinic crystal, potassium rare-earth tungstate

## Abstract

Monoclinic potassium rare-earth crystals are known as efficient materials for solid-state lasers and acousto-optic modulators. A number of specific configurations for acousto-optic devices based on those crystals have recently been proposed, but the acousto-optic effect of those crystals has only been analyzed fragmentarily for some interaction directions. In this work, we numerically searched for the global maxima of an acousto-optic figure of merit for isotropic diffraction in KGd(WO_4_)_2_ and KY(WO_4_)_2_ crystals. It was demonstrated that the global maxima of the acousto-optic figure of merit in those crystals occur in the slow optical mode propagating along the crystal’s twofold symmetry axis and in the acoustic wave propagating orthogonally, both for quasi-longitudinal and quasi-shear acoustic modes. The proposed calculation method can be readily used for the optimization of the acousto-optic interaction geometry in crystals with arbitrary symmetry.

## 1. Introduction

In the recent decade, there has been a growth of interest in the acousto-optic (AO) properties of biaxial crystals [1,2,3,4,5,6,7,8,9,10,11,12,13,14,15,16,17,18,19,20,21,22,23,24]. The main factors motivating the research in this area are (1) the good optical and acousto-optic properties of some biaxial crystals used in nonlinear optics and laser physics, and (2) a larger number of material constants in biaxial crystals as compared to uniaxial ones, which provides more useful configurations of AO interactions. Moreover, biaxial crystals are of special interest in AO research since they can provide unique configurations of the Bragg diffraction for laser beam deflection and spatial filtering [11,19,24].

In particular, potassium rare-earth tungstate crystals, also known as the KREW family, have recently been recognized as efficient crystal materials for applications in acousto-optics [2,3,4]. Those crystals belong to the monoclinic crystal system, which ensures a large number of independent elastic and photoelastic constants. Potassium gadolinium tungstate (KGW), KGd(WO${}_{4}$)${}_{2}$, and potassium yttrium tungstate (KYW), KY(WO${}_{4}$)${}_{2}$, are the most prominent crystals of the KREW family. Previous studies included measurements and computations of the AO figure of merit $M_{2}$ only for certain geometries of an AO interaction, with light propagating near the twofold symmetry axis of the crystal and ultrasound propagating on the symmetry plane [3,4,7,14,22]. A fair AO figure of merit in KREW crystals has been combined with a high laser-induced damage threshold [23,25]. This allowed for the design and fabrication of several experimental configurations of AO modulators (AOMs) and *Q*-switches for infrared (IR)-pulsed lasers [14,15,17,23]. Specific combinations of the acoustic and photoelastic properties of those crystals has prompted the creation of novel designs for AO devices, including spatial light modulators [18] as well as two-coordinate monolithic deflectors and polarization switches [22].

In this work, we explore the spatial distributions of an AO figure of merit $M_{2}$ in monoclinic KREW crystals with an arbitrary orientation of light and ultrasound under the assumption of a small-angle orthogonal diffraction. A procedure that can be used to search for the global maxima of the $M_{2}$ for isotropic AO diffraction in crystals with arbitrary symmetry is proposed and validated. Numerical results for KGW and KYW crystals are obtained and analyzed.

## 2. Materials and Methods

### 2.1. KREW Crystals’ Physical Properties

**Figure 1:**
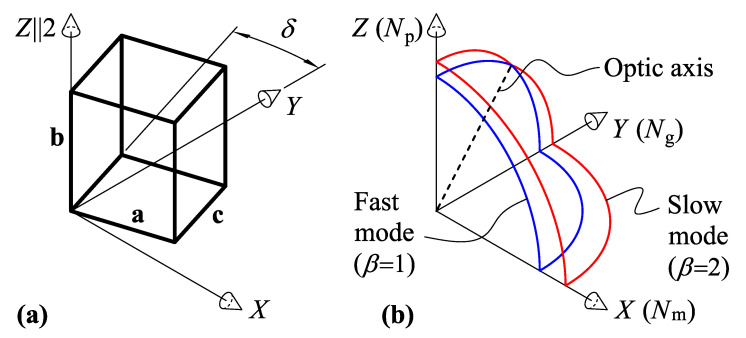
Dielectric properties of the KYW and KGW monoclinic crystals: (**a**) crystal’s unit cell; (**b**) refractive index surface cut-out. *Z* is the twofold symmetry axis; XY is the symmetry plane; optic axis (dashed line) is on the ZY plane.

KYW and KGW are monoclinic crystals of the 2/m point group. The orientation of the dielectric axes relative to the crystal’s unit cell is shown in Figure 1. We use the axes setting for monoclinic crystals, with the Z axis being parallel to the twofold symmetry axis of the crystal (b crystallographic axis and $N_{p}$ dielectric axis); *X* and *Y* are the dielectric axes $N_{m}$ and $N_{g}$, respectively.

The acoustic and photoelastic constants of KREW crystals have been measured by Mazur et al. [2,6]. The stiffness and photoelastic tensors, $c_{ijkl}$ and $p_{ijkl}$, are written in Voight’s notation. In monoclinic crystals, there are 13 independent stiffness coefficients $c_{qr}$:

(1) $$c_{qr}=\left(\begin{array}{cccccc} c_{11} & c_{12} & c_{13} & 0 & 0 & c_{16}\\ c_{12} & c_{22} & c_{23} & 0 & 0 & c_{26}\\ c_{13} & c_{23} & c_{33} & 0 & 0 & c_{36}\\ 0 & 0 & 0 & c_{44} & c_{45} & 0\\ 0 & 0 & 0 & c_{45} & c_{55} & 0\\ c_{16} & c_{26} & c_{36} & 0 & 0 & c_{66} \end{array}\right)$$

and 20 independent photoelastic coefficients $p_{qr}$:

(2) $$p_{qr}=\left(\begin{array}{cccccc} p_{11} & p_{12} & p_{13} & 0 & 0 & p_{16}\\ p_{21} & p_{22} & p_{23} & 0 & 0 & p_{26}\\ p_{31} & p_{31} & p_{33} & 0 & 0 & p_{36}\\ 0 & 0 & 0 & p_{44} & p_{45} & 0\\ 0 & 0 & 0 & p_{54} & p_{55} & 0\\ p_{61} & p_{62} & p_{63} & 0 & 0 & p_{66} \end{array}\right).$$

The values of the stiffness, photoelastic, and dielectric constants for the KGW crystal are listed in Appendix A.1, and those for the KYW crystal are in Appendix A.2.

### 2.2. Acousto-Optic Figure of Merit

The efficiency of the AO interaction in crystals depends on both acoustic and optical wave propagation directions with respect to the crystal axes. Any direction of the acoustic wave vector s is associated with three bulk acoustic wave (BAW) eigenmodes having orthogonal polarization vectors u and different phase velocities *v*. The exceptions are the acoustic axes, i.e., the directions corresponding to the degeneracy of two acoustic modes [21]. Any propagation direction of light m in a birefringent crystal is associated with two orthogonal eigenmodes that have polarization vectors d and refractive indices *n*. There are two types of AO diffraction: isotropic diffraction, which corresponds to the same polarization of incident and diffracted beams, and anisotropic diffraction, which corresponds to the orthogonal polarization of the beams.

Furthermore, we assume the isotropic diffraction type and remain within a small-angle approximation, i.e., that the optical wave normal vector m and the polarization vector d are the same for incident and diffracted waves. This approximation is valid for most of the configurations of isotropic diffraction, except for those where light propagates near the optic axis of a biaxial crystal. In the neighborhood of conical optic axes, the polarizations of eigenwaves d rapidly change with the wave normal direction m, and isotropic diffraction may be mixed with the anisotropic one [24]. In the other cases, i.e., those far from singular points of the refractive index surface and those in the typical range of ultrasound frequencies of the Bragg diffraction—from a few tens to a few hundred MHz—the double Bragg angle is on the order of $1^{{}^{\circ}}$, and the difference between polarization vectors d in the directions of incident and diffracted light is small. Thus, the small-angle approximation allows one to eliminate the ultrasound frequency from the problem parameters. In addition, the error of the experimental measurement of the photoelastic constants is more than 10% [6], therefore the error of $M_{2}\propto p_{\mathrm{eff}}^{2}$ can be estimated to be at 20 %.

**Figure 2:**
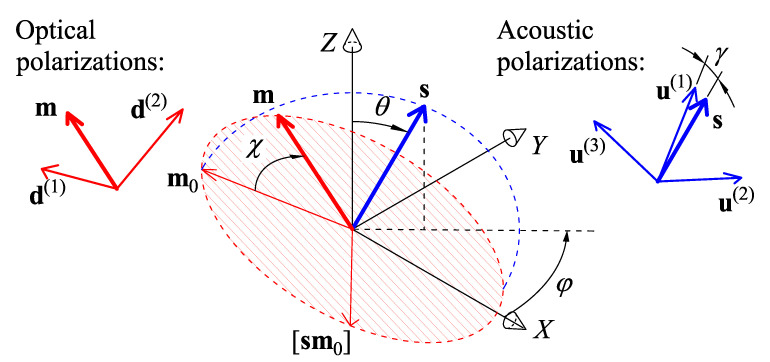
Spatial diagrams for $M_{2}$ calculations: a selection of acoustic and optical wave propagation directions and polarizations of optical and acoustic waves. Dashed plane is orthogonal to the acoustic direction unit vector s. The locus of optical wave normal vectors m for the given acoustic wave vector direction s is shown with hatching.

Figure 2 illustrates the orientation of the acoustic and optical wave vectors and polarizations. Standard procedures for calculating the optical and acoustic eigenmodes in crystals are described elsewhere [26,27]. Hereinafter, we sort the acoustic and optical modes of crystals from the fastest to the slowest, i.e., $v^{\left(1\right)}>v^{\left(2\right)}⩾v^{\left(3\right)}$ and $n^{\left(1\right)}⩽n^{\left(2\right)}$. The fastest acoustic mode $u^{\left(1\right)}$ is a quasi-longitudinal (QL) wave, and the other modes, $u^{\left(2\right)}$ and $u^{\left(3\right)}$, are quasi-shear (QS) waves. The polarization angle of acoustic waves is defined as $\gamma =∠(s,u^{\left(1\right)})$.

The direction of the acoustic wave vector is a function of two Euler angles, $\varphi \in [0,360^{{}^{\circ}}]$ and $\theta \in [0,90^{{}^{\circ}}]$, and is relative to the axes *Z* and *X*:

(3) s(φ,θ)={sinθcosφ,sinθsinφ,cosθ}.

Small-angle approximation is equivalent to orthogonal diffraction geometry, (ms)=0, so one can use the parametrization of m as follows:

(4) $$m\left(s,\chi \right)=m_{0}\cos\chi +\left[m_{0}s\right]\sin\chi ,$$

where the initial direction $m_{0}$ is orthogonal to the line of nodes:

(5) $$m_{0}\left(\varphi ,\theta \right)=\left\{-\cos\theta \cos\varphi ,-\cos\theta \sin\varphi ,\sin\theta \right\}.$$

For a certain interaction geometry, the effective photoelastic constant is expressed as follows [28]:

(6) $$p_{\mathrm{eff}}^{\left(\alpha ,\beta \right)}\left(s,m\right)=d_{i}^{\left(\beta \right)}d_{j}^{\left(\beta \right)}p_{ijkl}s_{k}u_{l}^{\left(\alpha \right)}$$

where α=1,2,3 is the acoustic mode number, β=1,2 is the optical mode number, and summation over repeated lower indices is assumed. In Equation (7), $d_{i}$ and $d_{j}$ are the components of the optical polarization eigenvector $d^{\left(\beta \right)}\left(m\right)$ corresponding to the β-th optical mode, and u(s) is the acoustic displacement vector corresponding to the α-th acoustic mode.

(7) $$M_{2}^{\left(\alpha ,\beta \right)}\left(s,m\right)=\frac{{n^{\left(\beta \right)}}^{6}{p_{\mathrm{eff}}^{\left(\alpha ,\beta \right)}}^{2}}{\rho {v^{\left(\alpha \right)}}^{3}}$$

Numerical maximum search was used to find the optimal propagation of the optical beam that maximizes $M_{2}$ for a given BAW propagation direction. The result of this search is the optical direction, expressed through angle χ:

(8) $$\chi_{\max}^{\left(\alpha ,\beta \right)}\left(s\right)=\underset{\chi \in [0,360^{{}^{\circ}}]}{\arg\;\max}M_{2}^{\left(\alpha ,\beta \right)}\left(s,m\left(s,\chi \right)\right)$$

The final result is the maximum figure of merit for a chosen acoustic direction s and combination of modes (α,β).

(9) $$M_{2\max}^{\left(\alpha ,\beta \right)}\left(\varphi ,\theta \right)=M_{2}^{\left(\alpha ,\beta \right)}\left(s,m\left(s,\chi_{\max}^{\left(\alpha ,\beta \right)}\right)\right)$$

In total, there are 6 combinations of optical and acoustic modes. Each combination was processed independently.

### 2.3. Software

The original software for computing $M_{2\max}$ was developed in the Fortran programming language. The initial data are the set of material constants (dielectric permittivity tensor $\varepsilon_{ii}$, stiffness $c_{pq}$ and photoelastic $p_{qr}$ matrices) defined in the crystals’ dielectric axes. The software is based on numerical techniques of linear algebra instead of analytical solutions. Therefore, it is universal and can be applied to crystals of any symmetry.

Standard methods for calculating the optical and acoustic eigenmodes in crystals were used for every acoustic wave normal vector s and optical wave normal vector m [26,27]. Then, Equations (6)–(9) were used for each possible pair of mode indices α and β. In the computations, the grid steps were $2^{{}^{\circ}}$ for φ and $1^{{}^{\circ}}$ for θ and χ.

## 3. Results

The results of the numerical simulations for KGW and KYW are $M_{2\max}^{\left(\alpha ,\beta \right)}$ and $\chi_{\max}^{\left(\alpha ,\beta \right)}$, as functions of the Euler angles φ and θ for three BAW modes (α=1,2,3) and two optical modes (β=1,2) of the crystals. Full numerical data on $\chi_{\max}$ and $M_{2\max}$ can be found in the related dataset in [29]. All diagrams are plotted in stereographic projection showing the upper hemisphere, $\theta \in [0,90^{{}^{\circ}}]$.

**Figure 3:**
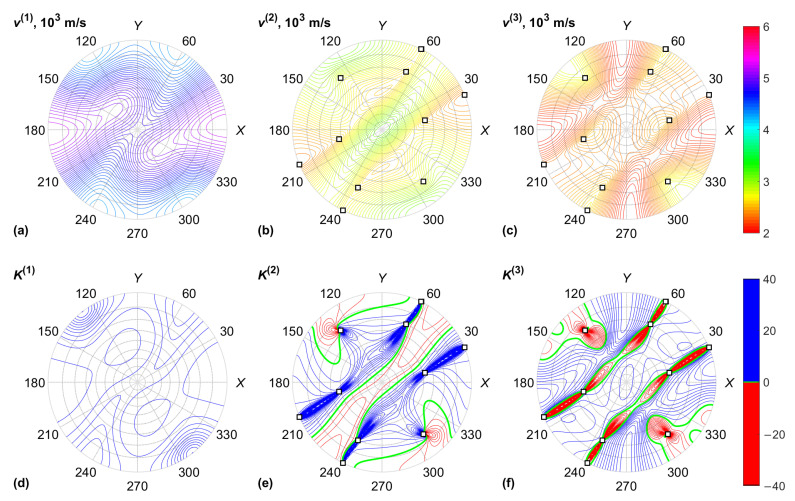
KGW crystal, acoustic properties: (**a**) QL BAW mode velocity; (**b**) fast QS BAW mode velocity; (**c**) slow QS BAW mode velocity; (**d**) QL BAW diffraction coefficient; (**e**) fast QS BAW diffraction coefficient; (**f**) slow QS BAW diffraction coefficient. Conical acoustic axes of the crystal are marked with squares.

**Figure 4:**
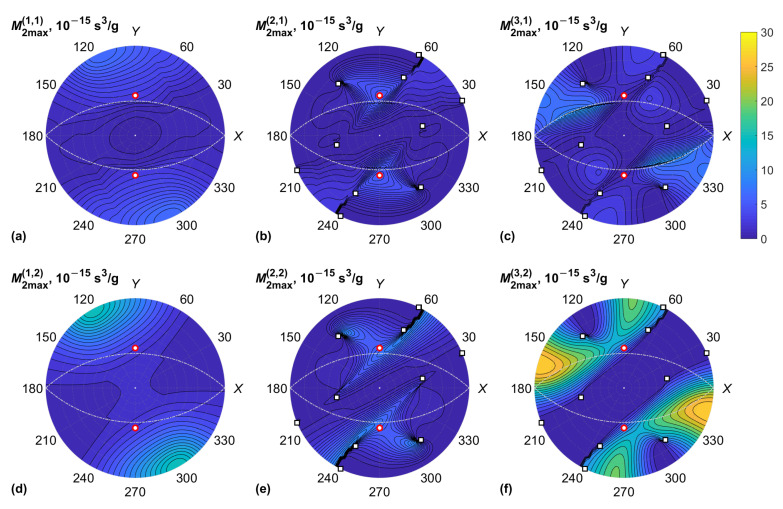
KGW crystal, maximum AO figure of merit, $M_{2\max}^{\left(\alpha ,\beta \right)}$ vs. BAW direction (φ,θ): (**a**) QL BAW mode (α=1), fast optical mode (β=1); (**b**) fast QS BAW mode (α=2), fast optical mode (β=1); (**c**) slow QS BAW mode (α=3), fast optical mode (β=1); (**d**) QL BAW mode (α=1), slow optical mode (β=2); (**e**) fast QS BAW mode (α=2), slow optical mode (β=2); (**f**) slow QS BAW mode (α=3), slow optical mode (β=2). Conical acoustic axes of the crystal are marked with squares; optic axes are marked with red circles; directions orthogonal to optic axes are shown with dash-dotted lines.

**Figure 5:**
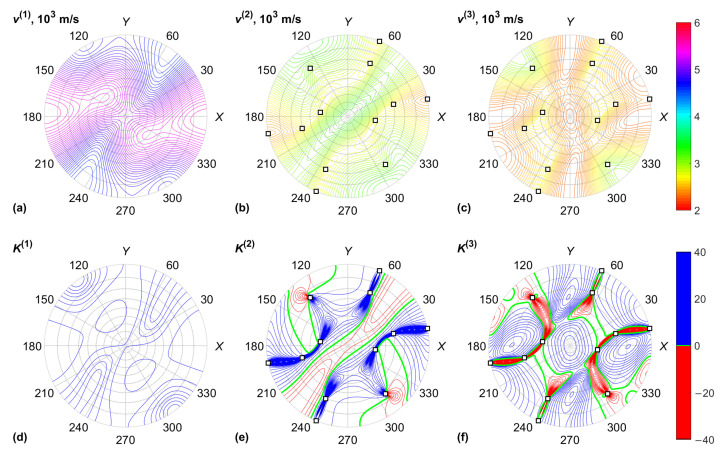
KYW crystal, acoustic properties: (**a**) QL BAW mode velocity; (**b**) fast QS BAW mode velocity; (**c**) slow QS BAW mode velocity; (**d**) QL BAW diffraction coefficient; (**e**) fast QS BAW diffraction coefficient; (**f**) slow QS BAW diffraction coefficient. Conical acoustic axes of the crystal are marked with squares.

**Figure 6:**
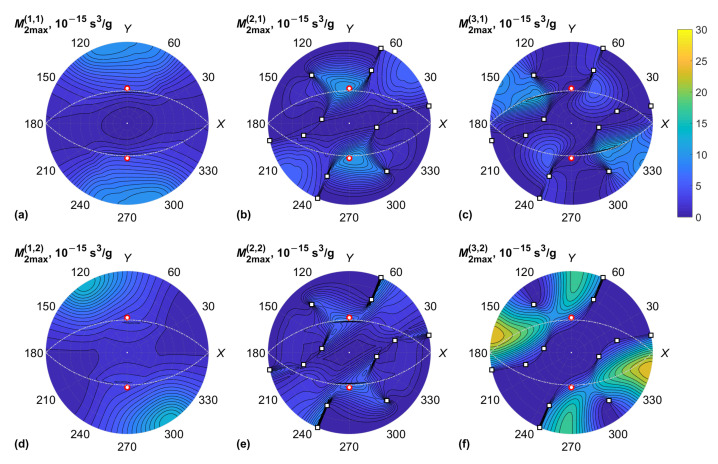
KYW crystal, maximum AO figure of merit: (**a**) QL BAW mode, fast optical mode; (**b**) fast QS BAW mode, fast optical mode; (**c**) slow QS BAW mode, fast optical mode; (**d**) QL BAW mode, slow optical mode; (**e**) fast QS BAW mode, slow optical mode; (**f**) slow QS BAW mode, slow optical mode. Conical acoustic axes of the crystal are marked with squares; optic axes are marked with red circles; directions orthogonal to optic axes are shown with dash-dotted lines.

The data for the KGW crystal are plotted in Figure 3 and Figure 4, including the acoustic properties of the crystal: BAW velocities $v^{\left(\alpha \right)}$ and normalized Gaussian curvatures $K^{\left(\alpha \right)}$ of the slowness surfaces. The Gaussian curvature is the product of two dimensionless BAW diffraction coefficients, which characterize the anisotropy of acoustic beam diffraction in crystals [30]. This coefficient tends to infinity in the neighborhood of conical acoustic axes. Similar results for the KYW crystal are plotted in Figure 5 and Figure 6.

Table 1 summarizes diffraction geometry parameters that maximize the AO figure of merit $M_{2}$ in KGW and KYW crystals. The table includes the direction angles φ and θ of the BAW, the optical rotation angle χ that defines the diffraction plane, and polarizations of the interacting beams. The data for the QS modes include the global maxima over both QS modes.

General features of $M_{2}$ data presented in Figure 4 and Figure 6 are the following: Firstly, the maxima of the figure of merit are associated with the minima of BAW velocity since $M_{2}\propto v^{-3}$. Secondly, rapid changes in $M_{2}$ for the QS BAW modes took place near the acoustic axes of the crystal and the directions of high acoustic anisotropy between them. The reason for this is the fast rotation of BAW polarization vectors $u^{\left(2\right)}$ and $u^{\left(3\right)}$ near the directions of high anisotropy [30]. Rapid changes in $M_{2\max}$ were also observed for some BAW directions orthogonal to the optic axes of the crystal since the optical wave normal vector m in this case crossed the optic axis associated with polarization singularity. However, we note that the small-angle approximation has a limited validity when light propagates near an optic axis because of the high optical anisotropy and singularity of the polarization field affecting the AO phase matching [24]. For this reason, the AO diffraction of light propagating near the optic axis of a biaxial crystal cannot be used for the design of AO modulators. On the other hand, it enables the design of unique types of AO devices for the deflection of light and processing vector beams [1,5,11,19]. In this case, the AO figure of merit should be calculated with respect to the actual frequency of ultrasound and the polarizations of optical eigenmodes.

## 4. Discussion

According to the plots in Figure 4 and Figure 6, the global maxima of the figure of merit $M_{2}$ occurred for the QS BAW mode propagating on the XY plane and which has a displacement orthogonal to the *Z* axis. The peaks of $M_{2}$ were higher for the slow optical mode (β=2). The global maxima were achieved at χ=0, which corresponds to the optical wave normal vector m parallel to the *Z* axis. To prove this, we selected the BAW propagation in the XY plane, i.e., $\theta =90^{{}^{\circ}}$, and φ is a variable; we then calculated the figure of merit $M_{2}\left(\chi \right)$ analytically.

Analytical expressions for $p_{\mathrm{eff}}$ can be readily derived for the case of χ=0, i.e., an optical wave propagating along the *Z* axis [22]:

(10) $$p_{\mathrm{ql}}^{\left(i\right)}=p_{i1}\cos\varphi \cos\left(\varphi +\gamma \right)+p_{i2}\sin\varphi \sin\left(\varphi +\gamma \right)+p_{i6}\sin\left(2\varphi +\gamma \right);$$

(11) $$p_{\mathrm{qs}}^{\left(i\right)}=-p_{i1}\cos\varphi \sin\left(\varphi +\gamma \right)+p_{i2}\sin\varphi \cos\left(\varphi +\gamma \right)+p_{i6}\cos\left(2\varphi +\gamma \right),$$

where i=1 for the fast optical mode with the polarization vector $d^{\left(1\right)}=\left\{1,0,0\right\}$, and i=2 for the slow optical mode with $d^{\left(2\right)}=\left\{0,1,0\right\}$. Equations (10) and (11) describe the interaction geometry with the optical beam propagating along the *Z* axis, i.e., at χ=0. These equations are also valid at i=3 for the fast optical mode propagating orthogonally to s on the XY plane, i.e., at $\chi =\pm 90^{{}^{\circ}}$. This mode has a polarization vector of $d^{\left(1\right)}=\left\{0,0,1\right\}$. The slow optical mode for this direction has a polarization of $d^{\left(2\right)}=\left\{\cos\varphi ,\sin\varphi ,0\right\}$ and the following effective photoelastic constants:

(12) $${\left.p_{\mathrm{ql}}\right|}_{\chi =90^{{}^{\circ}}}=p_{\mathrm{ql}}^{\left(1\right)}\cos^{2}\varphi +p_{\mathrm{ql}}^{\left(2\right)}\sin^{2}\varphi +p_{\mathrm{ql}}^{\left(6\right)}\sin2\varphi ;$$

(13) $${\left.p_{\mathrm{qs}}\right|}_{\chi =90^{{}^{\circ}}}=p_{\mathrm{qs}}^{\left(1\right)}\cos^{2}\varphi +p_{\mathrm{qs}}^{\left(2\right)}\sin^{2}\varphi +p_{\mathrm{qs}}^{\left(6\right)}\sin2\varphi .$$

To the best of our knowledge, the $p_{61}$, $p_{62}$, $p_{63}$, and $p_{66}$ constants have not yet been measured [6], and in all simulations, they were used with zero values.

**Figure 7:**
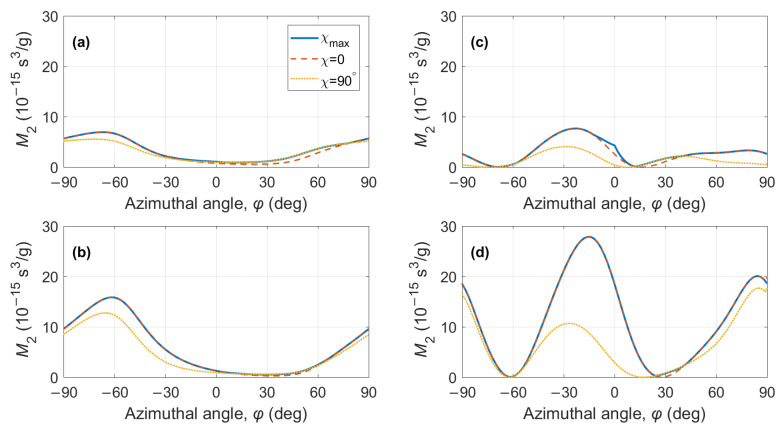
KGW crystal, AO figure of merit for acoustic propagation on the XY plane ($\theta =90^{{}^{\circ}}$): (**a**) QL BAW mode, fast optical mode; (**b**) QL BAW mode, slow optical mode; (**c**) QS BAW mode, fast optical mode; (**d**) QS BAW mode, slow optical mode. Solid lines—data from Figure 4; dashed and dotted lines—calculation according to Equations (10)–(13).

**Figure 8:**
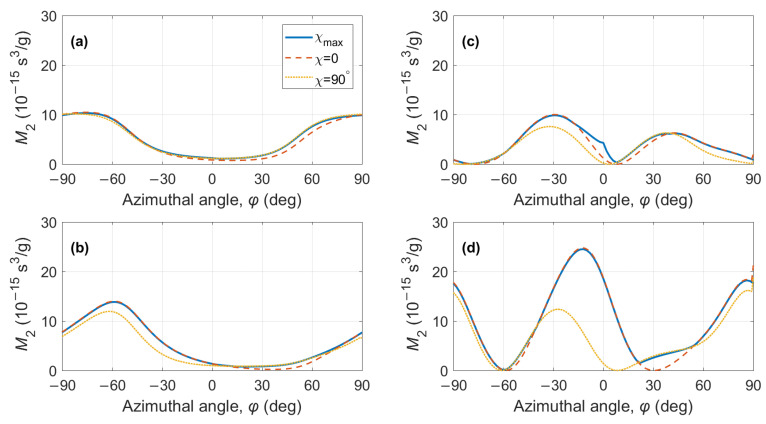
KYW crystal, AO figure of merit for acoustic propagation on the XY plane ($\theta =90^{{}^{\circ}}$): (**a**) QL BAW mode, fast optical mode; (**b**) QL BAW mode, slow optical mode; (**c**) QS BAW mode, fast optical mode; (**d**) QS BAW mode, slow optical mode. Solid lines—data from Figure 6; dashed and dotted lines—calculation according to Equations (10)–(13).

The maximum values $M_{2\max}$ are compared with the calculated data in Figure 7 and Figure 8. In panels (a) and (b), the maximum figure of merit was found for the QL BAW mode and compared to the calculations in Equations (10) and (12). In panels (c) and (g), the maximum figure of merit was found for both QS BAW modes, the fast and the slow, and then compared to the calculations from Equations (11) and (13).

## 5. Conclusions

We have reported a procedure that can be used to search for the global maxima of the AO figure of merit in crystals of lower symmetry classes. The procedure was able to find the optimal propagation direction of light and the corresponding figure of merit as the functions of the BAW propagation direction in crystals. We demonstrated this procedure with the use of two monoclinic crystals, KGW and KYW, which have previously shown high AO efficiency in certain configurations of AO interaction in the near-IR spectral region.

Our numerical simulations demonstrated that the global maxima of the figure of merit $M_{2}$ for isotropic diffraction in KGW and KYW crystals can be obtained for the BAW propagation on the XY plane and the optical propagation along the *Z* crystal axis. This conclusion is valid both for QL and QS BAW modes, and the highest $M_{2}$ values are produced for the slow optical mode polarized along the *Y* (i.e., $N_{g}$) dielectric axis. Nevertheless, we note that numerical data on $M_{2}$ in KREW crystals should be refined in the future since 8 out of the 20 photoelastic constants of these crystals have not been measured to date.

## Figures and Tables

**Table 1:** Maxima of the AO figure of merit in KGW and KYW crystals.

Parameter	KGW Crystal		KYW Crystal	
BAW mode, α	1	3	1	3
Polarization type and angle, γ	QL ($0.7^{{}^{\circ}}$)	QS ($6.5^{{}^{\circ}}$)	QL ($1.0^{{}^{\circ}}$)	QS ($4.7^{{}^{\circ}}$)
Velocity, *v* ($10^{3}$ m/s)	4.33	2.22	4.73	2.38
Azimuthal angle, φ	$-62^{{}^{\circ}}$	$-16^{{}^{\circ}}$	$-58^{{}^{\circ}}$	$-12^{{}^{\circ}}$
Polar angle, θ	$90^{{}^{\circ}}$	$90^{{}^{\circ}}$	$90^{{}^{\circ}}$	$90^{{}^{\circ}}$
Optical mode, β	2	2	2	2
Rotation angle, χ	0	0	0	0
Figure of merit, $M_{2}$ ($10^{-15}$ s${}^{3}$/g)	15.8	27.8	13.8	24.5

## Data Availability

The data presented in this study are openly available on Mendeley Data at http://dx.doi.org/10.17632/cg38s35c8d.1, accessed on 24 October 2022.

## Abbreviations

The following abbreviations are used in this manuscript:

AO	Acousto-optic
BAW	Bulk acoustic wave
IR	Infrared
KGW	KGd(WO${}_{4}$)${}_{2}$, potassium gadolinium tungstate
KREW	Potassium rare-earth tungstate
KYW	KY(WO${}_{4}$)${}_{2}$, potassium yttrium tungstate
QL	Quasi-longitudinal
QS	Quasi-shear

## Appendix A

### Appendix A.1

Material constants of the KGW crystal relevant to this research are the following: The crystal density is ρ=7.270 g/cm${}^{3}$. The elastic constants of the KYW crystal are as follows [2]:

(A1) $$c_{qr}=\left(\begin{array}{cccccc} 197.8 & 51.1 & 92.5 & 0 & 0 & -3.7\\ \; & 140.0 & 61.3 & 0 & 0 & -0.9\\ \; & \; & 171.7 & 0 & 0 & -3.0\\ \; & \; & & 64.0 & -12.1 & 0\\ \; & \; & & \; & 45.2 & 0\\ \; & \; & & \; & & 31.4 \end{array}\right)\cdot \mathrm{GPa}$$

The lower sub-diagonal part of the matrix is not shown because of its symmetry. The photoelastic tensor of the KGW crystal is the following [6]:

(A2) $$p_{qr}=\left(\begin{array}{cccccc} 0.11 & 0.23 & 0.14 & 0 & 0 & -0.053\\ 0.13 & 0.28 & 0.09 & 0 & 0 & -0.13\\ 0.13 & 0.23 & 0.04 & 0 & 0 & -0.025\\ 0 & 0 & 0 & p_{44} & p_{45} & 0\\ 0 & 0 & 0 & p_{54} & p_{55} & 0\\ p_{61} & p_{62} & p_{63} & 0 & 0 & p_{66} \end{array}\right)$$

Eight coefficients in the lower half of the matrix are not known. The optical properties of KGW are described with single-term, IR-corrected Sellmeier’s equations [31]:

(A3) $$\begin{array}{cc} \varepsilon_{11}=n_{m}^{2}= & 0.8753+3.0976\lambda^{2}/\left(\lambda^{2}-0.1554^{2}\right)-0.00042\lambda^{2};\\ \varepsilon_{22}=n_{g}^{2}= & 1.5157+2.6141\lambda^{2}/\left(\lambda^{2}-0.1733^{2}\right)+0.00183\lambda^{2};\\ \varepsilon_{33}=n_{p}^{2}= & 0.9927+2.8661\lambda^{2}/\left(\lambda^{2}-0.1523^{2}\right)-0.00139\lambda^{2}, \end{array}$$

which correspond to the following refractive indices at λ=1.06 μm: $n_{p}=\sqrt{\varepsilon_{33}}=1.979$, $n_{m}=\sqrt{\varepsilon_{11}}=2.010$, and $n_{g}=\sqrt{\varepsilon_{22}}=2.050$. The angle between the dielectric axis *Y* and the crystallographic axis c is $\delta =21.5^{{}^{\circ}}$. This angle was not explicitly used in the calculations, but it is necessary for recalculating the stiffness and photoelastic constants in different frames of reference.

### Appendix A.2

Material constants of the KYW crystal relevant to this research are the following: The crystal density is ρ=6.565 g/cm${}^{3}$. The elastic constants of the KYW crystal are the following [2]:

(A4) $$c_{qr}=\left(\begin{array}{cccccc} 202.2 & 51.0 & 90.9 & 0 & 0 & -3.3\\ \; & 150.6 & 60.9 & 0 & 0 & -2.7\\ \; & \; & 176.8 & 0 & 0 & -6.2\\ \; & \; & & 61.9 & -15.4 & 0\\ \; & \; & & \; & 41.8 & 0\\ \; & \; & & \; & & 33.5 \end{array}\right)\cdot \mathrm{GPa}$$

The photoelastic tensor of the KYW crystal is as follows [6]:

(A5) $$p_{qr}=\left(\begin{array}{cccccc} 0.12 & 0.33 & 0.17 & 0 & 0 & -0.04\\ 0.14 & 0.27 & 0.10 & 0 & 0 & -0.14\\ 0.15 & 0.35 & 0.05 & 0 & 0 & -0.02\\ 0 & 0 & 0 & p_{44} & p_{45} & 0\\ 0 & 0 & 0 & p_{54} & p_{55} & 0\\ p_{61} & p_{62} & p_{63} & 0 & 0 & p_{66} \end{array}\right)$$

Eight coefficients in the lower half of the matrix are not known, similar to thet KGW crystal. The optical properties of KYW are described with single-term, IR-corrected Sellmeier’s equations [31]:

(A6) $$\begin{array}{cc} \varepsilon_{11}=n_{m}^{2}= & 2.5253+1.4638\lambda^{2}/\left(\lambda^{2}-0.2083^{2}\right)-0.00237\lambda^{2};\\ \varepsilon_{22}=n_{g}^{2}= & 2.3951+1.7564\lambda^{2}/\left(\lambda^{2}-0.2025^{2}\right)-0.00226\lambda^{2};\\ \varepsilon_{33}=n_{p}^{2}= & 2.6986+1.1578\lambda^{2}/\left(\lambda^{2}-0.2127^{2}\right)-0.00215\lambda^{2}, \end{array}$$

which correspond to the following refractive indices at λ=1.06 μm: $n_{p}=\sqrt{\varepsilon_{33}}=1.975$, $n_{m}=\sqrt{\varepsilon_{11}}=2.011$, and $n_{g}=\sqrt{\varepsilon_{22}}=2.053$. The angle between the dielectric axis *Y* and the crystallographic axis c is $\delta =17.5^{{}^{\circ}}$.
